# Soluble tumor endothelial marker 1 in heart failure with reduced ejection fraction: A pilot study

**DOI:** 10.3389/fcvm.2022.1015471

**Published:** 2022-12-15

**Authors:** Wen-Han Feng, Po-Sheng Chen, Hsing-Chun Chung, Yi-Hsiung Lin, Yi-Heng Li

**Affiliations:** ^1^College of Medicine, Institute of Clinical Medicine, National Cheng Kung University, Tainan, Taiwan; ^2^Department of Internal Medicine, Kaohsiung Municipal Ta-Tung Hospital, Kaohsiung Medical University Hospital, Kaohsiung Medical University, Kaohsiung, Taiwan; ^3^Department of Internal Medicine, College of Medicine, National Cheng Kung University Hospital, National Cheng Kung University, Tainan, Taiwan; ^4^Division of Cardiology, Department of Internal Medicine, Kaohsiung Medical University Hospital, Kaohsiung Medical University, Kaohsiung, Taiwan; ^5^Center for Lipid Biosciences, Kaohsiung Medical University Hospital, Kaohsiung, Taiwan

**Keywords:** biomarkers, heart failure, TEM1, endomyocardial biopsy (EMB), cardiac remodeling

## Abstract

**Background:**

Tumor endothelial marker 1 (TEM1/CD248) is a transmembrane protein that expresses in mesenchymal lineage derived cells during embryogenesis and becomes undetectable in normal adults after birth. Re-expression of TEM1 is found in organ fibrosis, wound healing and cardiac remodeling indicating its potential role in heart failure (HF). The purpose of this study is to explore the role of soluble TEM1 (sTEM1) in patients with HF with reduced ejection fraction.

**Methods:**

We examined endomyocardial biopsy specimens from three HF patients and blood samples from 48 patients admitted for acute decompensated HF (age 72 years, men 61.7%). The expression of TEM1 in cardiac tissue and concentrations of sTEM1 in plasma were evaluated. Cultured rat cardiomyocytes (H9c2) and human cardiac fibroblasts (HCF) were stimulated with hypoxia or transforming growth factor beta (TGF-β) to observe the release of sTEM1 into culture media. The conditioned media of hypoxia-stimulated H9c2 cells was harvested and added into cultured cardiac fibroblast to evaluate its biological effect.

**Results:**

Immunofluorescence study of biopsy specimens from three HF patients showed TEM1 expression in cardiomyocytes and cardiac fibroblasts. The plasma level of sTEM1 was significantly higher in patients (0.90 ± 0.23 vs. 0.33 ± 0.10 ng/mL, *p* = 0.032) with LVEF ≤ 35% compared with those with LVEF 36–49%. The sTEM1 levels had correlations with HF biomarkers of cardiac fibrosis, including growth differentiation factor-15 (GDF-15) and galectin-3. There was a significant increase in sTEM1 levels in the cultured media of H9c2 and HCF after being stressed with hypoxia or TGF-β. The conditioned media derived from hypoxia-stimulated H9c2 cells significantly increased cell proliferation of cardiac fibroblasts. This effect was partially reversed by anti-TEM1 antibody.

**Conclusion:**

This pilot study demonstrated that cardiac TEM1 expression was upregulated in HF. The levels of sTEM1 were significantly higher in HF patients with LVEF ≤ 35% and correlated with other biomarkers of cardiac fibrosis. *In vitro* study proved that functional sTEM1 was released into cultured media after stressing cardiomyocytes and HCF.

## 1 Introduction

Heart failure (HF) is a clinical syndrome caused by structural or functional cardiac disorders that impair the ability of ventricle to fill with or pump blood. Cardiac remodeling is a compensatory process preceding the onset of HF when facing cardiac stress and involves a complex process of molecular, cellular and interstitial changes (1, 2). Cardiac fibrosis plays an important role in cardiac remodeling and contributes to the progression of HF (3, 4). Several novel circulating biomarkers that reflect some aspects of the cardiac remodeling process, such as cardiac stretch, cardiomyocyte stress, hypertrophy, myocardial fibrosis and inflammation, have been developed to predict the risk of HF development, progression and long-term prognosis (5, 6). However, the clinical usefulness of any one of the novel biomarkers in clinical practice is still questionable due to the biological complexity of cardiac remodeling and HF (7).

Tumor endothelial marker 1 (TEM1), also known as endosialin or CD248, is a cell-bound transmembrane glycoprotein that has been classified as a C-type lectin-like membrane receptor (8). TEM1 was first discovered on the cell surface of stroma cells in cancer (9). Later studies found TEM1 expression is restricted to mesenchymal lineage-derived cells during embryogenesis and its expression level becomes very low in normal adults (10, 11). In addition to cancer, re-upregulation of TEM1 has been found in renal fibrosis, hepatic fibrosis and wound healing (12–14). During hepatic injury, TEM1 expression is significantly increased in hepatic stellate cells, also known as perisinusoidal cells which are the pericytes found in the perisinusoidal space of the liver (13). Deletion of TEM1 demonstrated decreased proliferative response of hepatic stellate cells to platelet-derived growth factor (PDGF) stimulation and has less collagen production (13). During the process of wound healing, TEM1 expression was highly upregulated in myofibroblasts (14). The fibroblast activation and collagen deposition in granulation tissues were attenuated and wound healing was retarded in TEM1-deleted mice (14). Expression of TEM1 in these pathological conditions suggests that TEM1 plays roles in tissue remodeling and repairing process. The protein structure of TEM1 includes an extracellular N-terminal C-type lectin domain (D1), a sushi domain (D2), an epidermal growth factor (EGF) domain (D3), a mucin-like region (D4), a transmembrane domain (D5), and a cytoplasmic tail (D6) (15). Like many other transmembrane proteins, the extracellular domains (D1–4) of TEM1 may be shed from the cell membrane to become soluble form and released into systemic circulation. Soluble TEM1 (sTEM1) still carries distinct protein functions and works in an endocrine or paracrine way (8, 16). So far, very little did we know about the role of TEM1 in HF. Our previous study demonstrated that TEM1 expression was increased in cardiomyocyte and cardiac fibroblasts in HF and stress with doxorubicin or hypoxia induced TEM1 expression in cultured cardiomyocytes (17). After proving the increased cell expression of TEM1 in HF, we focused on the role of sTEM1 in HF in the current study. This pilot study is a prove of concept research that we hypothesized that the TEM1 expressed in cardiac cells in HF may be released to form sTEM1 which can be detected in the peripheral circulation of HF patients. We examined the plasma levels of sTEM1 in HF patients and evaluated its relation with other circulating biomarkers of HF.

## 2 Materials and methods

### 2.1 Human study

#### 2.1.1 Study design

First, we evaluated the expression of TEM1 in myocardial specimens taken from HF patients by endomyocardial biopsy [IRB approved number: KMUHIRB-E(I)-20200051]. HF patients were admitted to the hospital for evaluation and treatment. Left ventricular ejection fraction (LVEF) was determined by echocardiography during admission. If clinically necessary, endomyocardial biopsy was performed to confirm the etiology of HF during hospitalization. The biopsy procedure was performed *via* the right internal jugular vein approach with a flexible bioptome device (Cordis Corp.). Under fluoroscopic guidance, the bioptome device was advanced into the right ventricle and biopsy of the right ventricular septum for 3–5 specimens was performed. Second, we used western blot to detect the presence of sTEM1 in the peripheral circulation of HF patients. Human blood samples were collected from HF patients during their admissions [IRB approved number KMUHIRB-E(I)-20180287]. Blood samples taken from two normal individuals (the major investigators of this study, Dr. Feng and Dr. Li) were used as control. The two normal individuals are completely healthy, without any systemic diseases and not taking any medication on the day when drawing the blood. Third, after proving that sTEM is present in the peripheral circulation of HF patients, we performed a larger cohort study to enroll patients who were admitted for acute decompensated HF with reduced LVEF and survived to discharge. All patients were treated by the attending cardiologists according to the current HF guideline. At the time of discharge when their clinical conditions improved, the blood samples were taken from these patients. The plasma level of sTEM1 and other HF biomarkers, including B-type natriuretic peptide (BNP), suppression of tumorigenicity 2 (ST2), growth differentiation factor (GDF-15), galectin-3, and inflammatory biomarkers, including interleukin-6 (IL-6), tumor necrosis factor-α (TNF-α) were measured with commercialized enzyme-linked immunosorbent assay (ELISA) according to the manufacturer’s instruction (sTEM1 from Cusabio and all others from R&D).

#### 2.1.2 Immunofluorescence staining

Normal human right ventricular tissues purchased from US Biomax, Inc., Derwood, USA (category number: HuFPT061) were used as control. We used immunofluorescence study to detect TEM1 expression in the myocardial specimens. The biopsy specimens from the HF patients and the purchased normal samples were stained with identical experimental procedures. For double immunofluorescent staining, the sections were stained with primary antibodies for TEM1 (1:1,000, ATLAS) and troponin (cardiomyocyte marker, 1:2,000 Abcam) or TEM1 (1:1,000, ATLAS) and fibroblast activation protein (FAP, cardiac fibroblast marker, 1:1,000 Abcam). Fluorescent secondary antibodies were goat anti-mouse IgG Alexa Fluor 546 and goat anti-rabbit IgG Alexa Fluor 488. Nuclei were counterstained with 4′, 6-diamidino-2-phenylindole (DAPI, Sigma). The specimens were evaluated by Nikon TE2000 confocal microscope. The immunofluorescence staining were quantified by Image J (Version Java 1.8.0_172 64-bit, National Institutes of Health, Bethesda, USA). We measured the areas of positive staining of the overlapping positions of two antibodies (TEM1 and troponin or TEM1 and FAP) in randomly selected 4 sections from each HF patient and normal control. The average value of the 12 sections of the 3 HF patients was compared with the normal control.

### 2.2 *In vitro* study

#### 2.2.1 Cell culture

The experiments used two types of cardiac cells: H9c2 cells and human cardiac fibroblasts (HCF). H9c2 cells were purchased from the Food Industry Research and Development Institute, Hsinchu, Taiwan. They are embryonic rat heart-derived cardiomyoblast and are a commonly used cell model of cardiomyocytes. HCF were purchased from the company (Sigma-Aldrich). The cells were cultured in Dulbecco’s modified Eagle’s medium or cardiac fibroblast growth medium containing 1% penicillin/streptomycin under an atmosphere with humidified 5% CO_2_ at 37^°^C. When adherent cells reached 80% confluence, the cells were used for stress experiments.

#### 2.2.2 Stress experiment

We stressed the H9c2 cells and HCF with hypoxia or pro-fibrotic mediator, transforming growth factor beta (TGF-β). The release of sTEM1 into cultured media was evaluated with western blot. In the hypoxia model, the cells were placed in a hypoxic incubator prefilled with 94% nitrogen gas, 5% CO_2_ and with only 1% oxygen for 24 h. In the pro-fibrotic mediator stimulation, 10 ng/mL TGF-β (R&D) was added to treat the cells. After hypoxia or TGF-β treatment, the media were harvested in 0.5, 2, 4, 8, 16, and 24 h after the treatment and the presence of sTEM1 in the media were determined with western blotting.

#### 2.2.3 Western blotting

The culture media were harvested, centrifuged, and then concentrated with an ultrafiltration system (Amicon Ultracel 30K, Millipore, Bedford, MA). Total protein (120 μg) from each sample were loaded into SDS-polyacrylamide gel. The separate proteins were transferred to a polyvinylidene difluoride membrane (Bio-Rad), blocked with 5% skim milk, and incubated with rabbit monoclonal anti-TEM1 antibody (Proteintech) at 4°C for overnight. A chemiluminescence reagent (Millipore) was used to detect the protein band and AlphaImager 2,200 digital imaging system was used to quantify the bands’ intensities.

#### 2.2.4 Conditioned media effect

We assessed the biological effect of conditioned media derived from hypoxia-stimulated H9c2 cells. After 24-h hypoxia treatment, the conditioned media of H9c2 cells were harvested and concentrated. We used the conditioned media to treat cultured cardiac fibroblasts and evaluated cell proliferation with 5-bromo-2-deoxyuridine (BrdU) assay. After receiving conditioned media treatment for 24 h, 10 mM BrdU was added and followed by incubation for 2 h. The nuclear incorporation of BrdU was measured using a cell proliferation ELISA kit (Roche Diagnostics, Mannheim, Germany). We also adding anti-TEM1 antibody (Proteitech) to see if it could block the conditioned media effect.

### 2.3 Statistical analysis

Continuous variables were presented as means ± standard errors and categorical variables as numbers and percentages. Comparisons between the groups were made by Wilcoxon rank sum test. Correlation analyses were performed with Spearman rank correlation test. All statistical analyses were performed using SPSS 22 (SPSS Inc., Chicago, IL, USA). A *p*-value < 0.05 is considered to be statistically significant.

### 2.4 Study approval

All human studies were performed in accordance with the World Medical Association Declaration of Helsinki. The study protocols were approved by the institutional review board (IRB) of the Kaohsiung Medical University Hospital, Kaohsiung, Taiwan and informed consents were obtained from the study participants [IRB approved number: KMUHIRB-E(I)-20200051, KMUHIRB-E(I)-20180287].

## 3 Results

### 3.1 Human study

#### 3.1.1 TEM1 in myocardial specimens and blood

**FIGURE 1:**
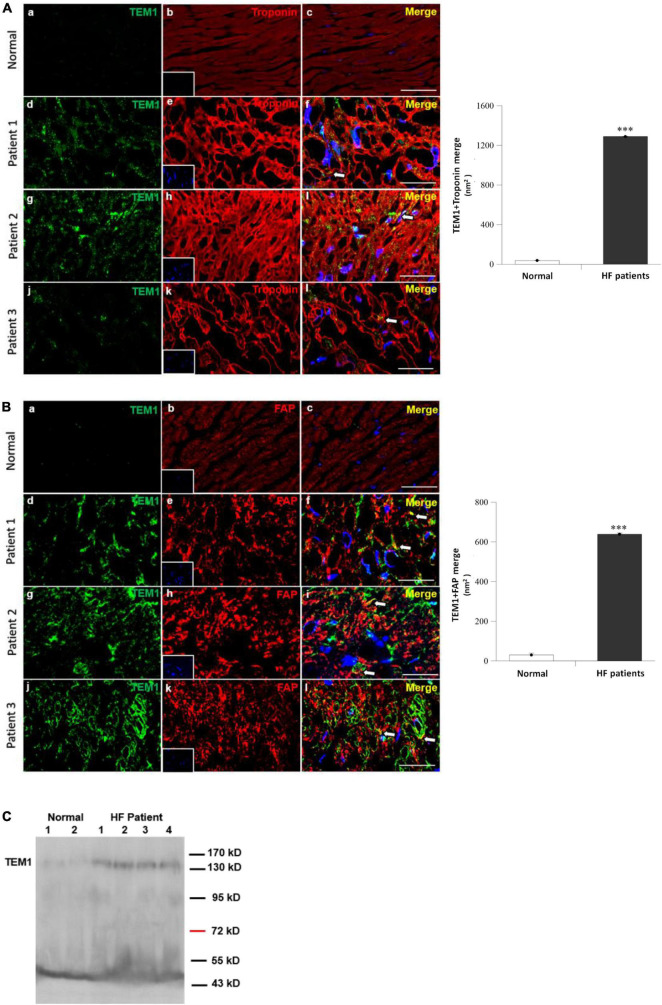
Tumor endothelial marker 1 in human heart failure **(A,B)**. Immunofluorescence staining of TEM1 in normal right ventricular tissues and three patients with heart failure received endomyocardial biopsy. Representative images show immunostaining for TEM1 (green) and troponin (red, cardiomyocyte marker) **(A)** or TEM1 (green) and fibroblast-activation protein (red, FAP, cardiac fibroblast marker) **(B)**. Nucleus was stained with DAPI (blue). Arrows indicate the TEM1 staining (merge) in cells. Scale bar = 25 μm. The area of positive TEM1 staining (merge) in randomly selected 4 sections in each patient and purchased samples were measured. The average value of the 12 sections of the three heart failure patients was compared with the control of normal ventricular tissue ^***^p < 0.001. Picture **(a–c)**: normal right ventricular tissue, picture **(d–f)**: patient 1, picture **(g–i)**: patient 2, and picture **(j–l)**: Patient 3. **(C)** Detection of sTEM1 in human plasma with western blotting in 4 heart failure patients and 2 normal healthy individuals.

Endomyocardial biopsy was performed in three patients with HF and reduced LVEF who were admitted for further diagnosis of HF etiology. The clinical characteristics of the 3 patients were shown in Table 1 and all of them had severely depressed LVEF (16.8, 24.5, and 15.4%, respectively). The final diagnoses of their etiologies of HF were ischemic cardiomyopathy in patient 1, dilated cardiomyopathy in patient 2, and acute myocarditis in patient 3. Double immunofluorescence staining of the myocardial specimens exhibited TEM1 staining in both cardiomyocytes (Figure 1A) and cardiac fibroblasts (Figure 1B) in the three patients. There was almost no TEM1 expression in normal human ventricular tissue. The fluorescence intensity of TEM1 expression was significantly higher in the HF patients compared with normal ventricular tissue after quantification. Western blot could detect the presence of sTEM1 with molecular weight around 130–160 KDa in the peripheral circulation of these three HF patients (Patient 1–3, Figure 1C). In another HF patient (LVEF 18.5%), sTEM1 in blood could also be detected by western blot (patient 4, Figure 1C). TEM1 was almost undetectable in the 2 healthy normal controls (normal 1 and 2, Figure 1C).

**TABLE 1:** Clinical characteristics of the three heart failure patients who received endomyocardial biopsy.

	Patient #1	Patient #2	Patient #3
Age (years)	78	59	71
Sex	Male	Male	Male
Rhythm	Sinus rhythm	Sinus rhythm	Sinus rhythm
**Echocardiographic findings**			
LVEDD (mm)	56.2	80.3	62.1
LVESD (mm)	51.9	68.0	57.7
LVEF (%)	16.8%	24.5%	15.4%
LAD (mm)	44.0	52.8	37.0
Coronary artery stenosis	Three-vessel disease	Normal coronary artery	Normal coronary artery
Final diagnosis	Ischemic cardiomyopathy	Dilated cardiomyopathy	Acute myocarditis

LAD, left atrial dimension; LVEDD, left ventricular end-diastolic dimension; LVEF, left ventricular ejection fraction; LVESD, left end-systolic dimension.

#### 3.1.2 Levels of sTEM1 in HF patients

**FIGURE 2:**
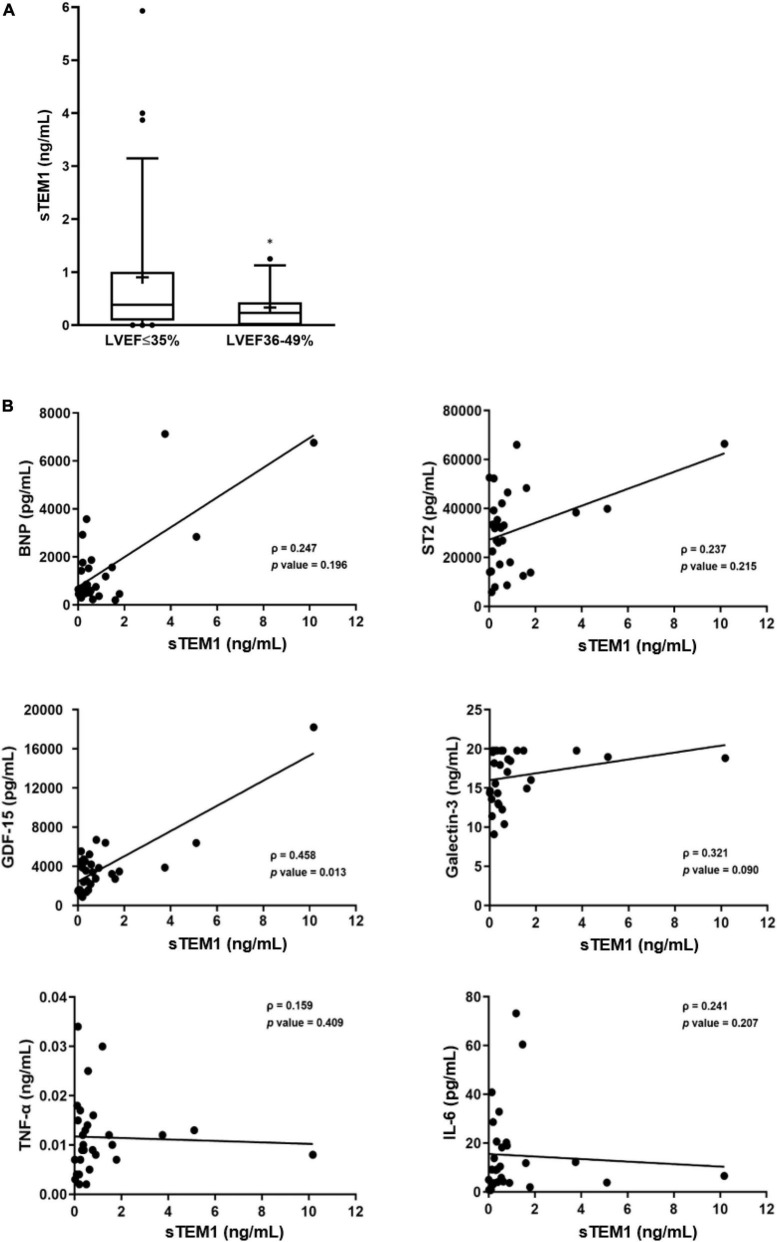
Soluble TEM1 in human heart failure. **(A)** Higher levels of sTEM1 in heart failure with severely depressed left ventricular ejection fraction *p < 0.05. **(B)** Correlations of sTEM1 with biomarkers of heart failure and inflammation in patients with heart failure. BNP, B-type natriuretic peptide; GDF, growth differentiation factor; ST2, suppression of tumorigenicity 2; TNF-α, tumor necrosis factor-α; IL-6, interleukin-6.

After proving TEM1 expression in cardiac cells and presence of sTEM1 in the peripheral circulation, we performed a cohort study and consecutively included 48 patients admitted with HF and reduced LVEF. Table 2 shows the clinical characteristics and medical therapies in these patients. There were 33 patients with severely depressed LVEF (≤ 35%) and 15 patients with mildly depressed LVEF (36–49%). The baseline characteristics of the patients in the two groups were similar. About half the patients had a history of diabetes, more than 50% of patients had previous myocardial infarction, and about 40% had atrial fibrillation. The plasma levels of BNP and sTEM1 was significantly higher in the severely depressed LVEF group (BNP, 3929.4 ± 4268.1 vs. 1882.5 ± 1181.8 pg/mL; sTEM1, 0.90 ± 1.34 vs. 0.33 ± 0.39 ng/mL, all *p* < 0.05) (Figure 2). We examined the correlation of sTEM1 with other HF biomarkers (BNP, ST-2, GDF-15, galectin-3) and pro-inflammatory biomarkers (IL-6, TNF). There was no significant correlation between sTEM1 and BNP and ST2. But sTEM1 had a significant correlation with GDF-15 (ρ = 0.458, *p* = 0.013), and a borderline correlation with galectin-3 (ρ = 0.321, *p* = 0.090). There was no significant correlation between sTEM1 and pro-inflammatory biomarkers, TNF-α and IL-6.

**TABLE 2:** Baseline characteristics and soluble TEM1 levels in HF patients.

	HF with LVEF ≤ 35% (*n* = 33)	HF with LVEF 36–49% (*n* = 15)	*P*-value
Age (years)	71.2 ± 2.3	72.3 ± 3.8	0.803
Male, *n* (%)	19 (57.6)	10 (66.7)	0.551
LVEF (%)	26.8 ± 0.9	41.5 ± 0.7	*p* < 0.001
Serum creatinine (mg/dL)	1.51 ± 0.24	1.34 ± 0.15	0.546
eGFR (mL/min/1.73 m^2^)	56.6 ± 5.1	58.1 ± 7.8	0.869
Previous myocardial infarction	19 (57.6)	10 (66.7)	0.551
Atrial fibrillation	14 (42.4)	6 (40.0)	0.875
Diabetes mellitus	18 (54.5)	7 (46.7)	0.613
Hypertension	27 (81.8)	12 (80.0)	0.881
Dyslipidemia	20 (60.6)	10 (66.7)	0.688
LDL-C (mg/dL)	106.1 ± 8.1	110.9 ± 7.8	0.721
Smoker	11 (33.3)	3 (20.0)	0.346
**Medications**			
Renin–angiotensin inhibitors	31 (93.9)	12 (80.0)	
Beta-blockers	29 (87.9)	12 (80.0)	
Mineralocorticoid receptor antagonists	20 (60.6)	6 (40.0)	
Soluble TEM1 (ng/mL)	0.90 ± 0.23	0.33 ± 0.10	0.032
BNP (pg/mL)	3929.4 ± 754.6	1882.5 ± 315.9	0.016

BNP, B-type natriuretic peptide; eGFR, estimated glomerular filtration rate; HF, heart failure; LVEF, left ventricular ejection fraction; LDL-C, low density lipoprotein cholesterol; TEM1, tumor endothelial marker 1.

### 3.2 *In vitro* study

#### 3.2.1 Release of TEM1 from the cells

**FIGURE 3:**
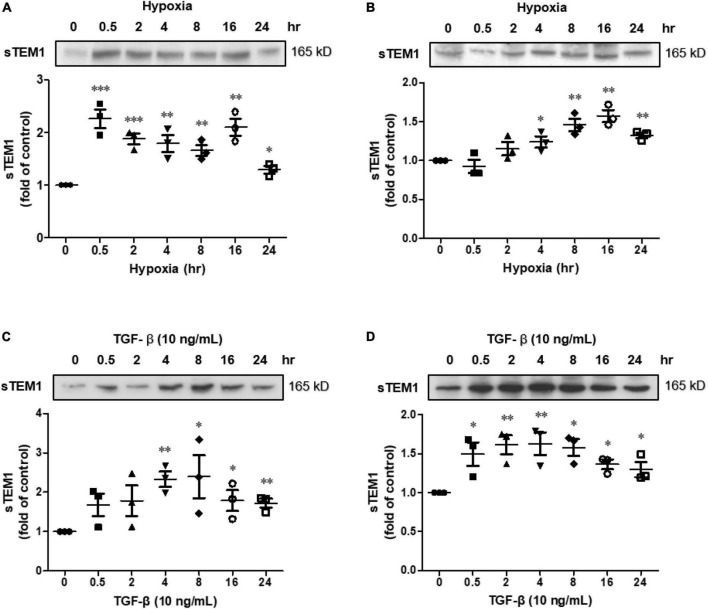
Soluble TEM1 in the cultured media. Soluble TEM1 in the conditioned media of cultured H9c2 cells **(A)** and cardiac fibroblasts **(B)** exposed to hypoxia incubator prefilled with 1% oxygen for 24 h. Soluble TEM1 in the conditioned media of cultured H9c2 cells **(C)** and cardiac fibroblasts **(D)** treated with pro-fibrotic mediator, TGF-β. The expression levels were analyzed by western blot and expressed as a ratio to baseline without hypoxia or TGF-β *n* = 3. *p < 0.05, ^**^p < 0.01 and ^***^p < 0.001 compared to normal at time 0.

Hypoxia was used to stress the cultured H9c2 cells and cardiac fibroblasts. The culture media were harvested and the presence of sTEM1 in the media was examined. After treating the cultured H9c2 cells with hypoxia, the presence of sTEM1 was detectable in the culture media by western blotting (Figure 3). The H9c2 cells exposed to hypoxia immediately released a significant amount of sTEM1 into the media. The sTEM1 levels in the media of the stressed cells increased significantly at 0.5 h after treatment and persisted for 24 h (Figure 3A). Compared to the baseline, the maximal increase of sTEM1 in the media after exposing the cells to hypoxia (2.49 ± 0.02 fold) occurred at 0.5 hr after treatment. Then, the sTEM1 levels decreased thereafter, but the sTEM1 levels in the media of the stressed cells were higher than those without stress at all-time points from 0.5 to 24 h. When using cardiac fibroblasts to repeat the same experiments, we also found sTEM1 was detectable in the culture media (Figure 3B). However, the degree of the sTEM1 level increase was smaller than that observed in the H9c2 cells. Compared to the baseline, the release of sTEM1 increased progressively from 0.5 to 24 h and the maximal release of sTEM1 in the media from cardiac fibroblasts after treating hypoxia was 1.22 ± 0.01 fold at 16 h after hypoxia treatment. In human study, we found increased sTEM1 levels had correlations with cardiac fibrotic biomarkers, GDF-15 and galectin-3. We evaluated the influence of pro-fibrotic mediator, TGF-β, on the sTEM1 release. Similar to hypoxic stimulation, the pro-fibrotic mediator, TGF-β could also induce the release of sTEM1 into the media from cultured cardiomyocytes (Figure 3C) and cardiac fibroblasts (Figure 3D).

#### 3.2.2 Conditioned media effect

**FIGURE 4:**
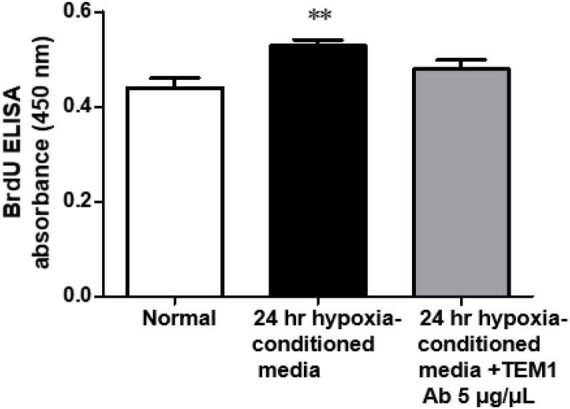
Biological effect of conditioned media. The conditioned media derived from hypoxia-stimulated H9c2 cells significantly increased the cell proliferation of cardiac fibroblasts and this effect was partially reduced with anti-TEM1 antibody *n* = 4. ^**^p < 0.01 compared to normal without adding conditioned media.

In order to prove the biological effects of sTEM1, we assessed the effect of conditioned media derived from hypoxia-stimulated H9c2 cells on cell proliferation. Figure 4 shows the conditioned media significantly increased the cell proliferation of cardiac fibroblasts. This effect was partially reduced after adding anti-TEM1 antibody.

## 4 Discussion

Our study demonstrated that TEM1 expression can be found in cardiomyocytes and cardiac fibroblasts in HF patients with reduced LVEF. The levels of sTEM1 can be detected in the peripheral circulation. A higher level of sTEM1 was found in those with poorer LVEF and correlated with other HF biomarkers, GDF-15 and galectin-3.

Our previous study showed that TEM1 expression was increased in cultured cardiomyocytes after doxorubicin or hypoxia treatment (17). Similar to the biological natures of many other transmembrane proteins, the extracellular domains of TEM1 (D1-4) can be broken and released from cell membrane into systemic circulation. A previous study demonstrated that sTEM1 with molecular weight ranging from 120 to 150 kDa could be found in systemic circulation in patients with colorectal cancer (16). In this pilot study, we first showed that sTEM1 levels in the systemic circulation of HF patients could be detected by western blot and ELISA. The sTEM1 levels were higher in the severely depressed LVEF group. Novel biomarkers are used increasingly in the evaluation of patients with HF. Since the pathophysiology of HF involves a complex interplay and changes between cardiac fibrosis, wall stress/stretch, inflammation, and neurohormones, the biomarkers should reflect one of these pathophysiologic mechanisms in HF. We found sTEM1 was mainly correlated with GDF-15 and galectin-3, but not BNP, a wall stretch marker, nor pro-inflammatory biomarkers of TNF-α and IL-6. GDF-15 and galectin-3 are associated with tissue fibrosis and inflammation, and both markers predict the prognosis of HF (18, 19). GDF-15 is a member of the transforming growth factor β (TGF-β) cytokine family and has been proposed as a marker of cardiac fibrosis (20, 21). Galectin-3 is a beta-galactoside-binding lectin that is also currently viewed as a novel biomarker for cardiac fibrosis and adverse cardiac remodeling in HF (22, 23). Previous studies have already demonstrated the important role of TEM1 in renal and hepatic fibrosis (12, 13). Our study indicated that TEM1 might involve in the progress of cardiac remodeling and cardiac fibrosis (17). We assume that upregulation of TEM1 in heart and increased sTEM1 levels is a compensatory mechanism in patients with HF in order to counteract cardiomyocyte damage and maintain adequate left ventricular function. The current pilot study carried an implication that sTEM1 may be a novel biomarker of severe HF, but further studies are needed to prove its clinical usefulness in evaluating the prognosis of HF and its impact on clinical outcomes.

Cardiomyocytes and cardiac fibroblasts are the major cell types in the mammalian heart (3, 24). In cardiac specimens from HF patients, both cell types expressed TEM1. Therefore, we used both cells to perform the *in vitro* study. The results indicated that the extracellular domains of TEM1 could be released to cultured media and become sTEM1 because the molecular weight of soluble TEM1 detected by western blot was smaller than the full-length TEM1. The sTEM1 levels were significantly increased after hypoxia in both cultured cardiomyocytes and cardiac fibroblasts. Cardiomyocytes had a more rapid response after stimulation than cardiac fibroblasts. The degree of sTEM released in the culture media was also more prominent in cardiomyocytes than in cardiac fibroblasts (2.49 ± 0.02 vs. 1.22 ± 0.01 fold of increase). Although cardiomyocytes carry a faster and more prominent response, cardiac fibroblasts seem to have a gradual-onset and more sustained effect of releasing sTEM1. These findings implied that TEM1 plays a role in the pathophysiologic response to injury both in cardiomyocytes and cardiac fibroblasts. Both cell types contribute to the release of sTEM1. Whether the concentration of sTEM1 in peripheral circulation could reflect the TEM1 expression in cardiac cells is still not clear. A previous study demonstrated that circulating concentrations of galactin-3 did not reflect the endomyocardial expression levels of galectin-3 (22). It is also unknown whether TEM1 expression in organs other than the heart could contribute to the systemic concentration of sTEM1 during HF. There were other limitations in our study. We analyzed the expression of TEM1 in cardiac specimens with immunofluorescence study. Because of the limited amount of tissue obtained from endomyocardial biopsy, it was not possible to perform western blot to prove the increased TEM1 expression in biopsy specimens. Second, the case numbers of HF included in this study were small. Because this is a pilot study and endomyocardial biopsy is an invasive procedure with risks of severe complications, we decided to enroll three patients to test the hypothesis first. Based on these findings in this pilot study, a further larger study that enrolls more HF patients is necessary to confirm the role of sTEM1 in HF. Third, we did not have the sTEM1 levels in enough numbers of normal healthy controls. We also did not know if there is any advantage of sTEM1 over other biomarkers in patients with HF. Finally, the mechanistic details about the role of TEM1 in HF and the potential influence of different etiologies of HF on sTEM1 levels also need further investigation.

## 5 Conclusion

Our pilot study showed that, in patients with HF, the levels of sTEM1 is detectable in the peripheral blood and had correlation with other HF biomarkers of fibrosis implying that TEM1 might have similar pathophysiological roles in HF. The expression of TEM1 is upregulated in cardiomyocytes and cardiac fibroblasts after stimulation and can be released from the cells into culture media.

## Data availability statement

The original contributions presented in this study are included in the article/supplementary material, further inquiries can be directed to the corresponding author.

## Ethics statement

The studies involving human participants were reviewed and approved by the Kaohsiung Medical University Hospital. The patients/participants provided their written informed consent to participate in this study.

## Author contributions

W-HF and Y-HeL: conceptualization and writing—original draft. W-HF, P-SC, H-CC, and Y-HsL: data collection, formal analysis, and investigation. Y-HeL: methodology. H-CC: project administration. Y-HeL: supervision and writing—review and editing. All authors read and agreed to the published version of the manuscript.
